# Assessing methods for measurement of clinical outcomes and quality of care in primary care practices

**DOI:** 10.1186/1472-6963-12-214

**Published:** 2012-07-23

**Authors:** Michael E Green, William Hogg, Colleen Savage, Sharon Johnston, Grant Russell, R Liisa Jaakkimainen, Richard H Glazier, Janet Barnsley, Richard Birtwhistle

**Affiliations:** 1 Department of Family Medicine, Queen’s University, Kingston, Ontario, Canada; 2 Department of Community Health and Epidemiology, Queen’s University, Kingston, Ontario, Canada; 3 Centre for Health Services and Policy Research, Queen’s University, Abramsky Hall, 3rd Floor, 21 Arch Street, Kingston, Ontario, K7L 3N6, Canada; 4 Centre for Studies in Primary Care, Queen’s University, Kingston, Ontario, Canada; 5 Institute for Clinical Evaluative Sciences, Toronto, Ontario, Canada; 6 Department of Family Medicine, University of Ottawa, Ottawa, ON, Canada; 7 CT Lamont Primary Care Research Centre, Ottawa, Ontario, Canada; 8 Southern Academic Primary Care Research Unit, School of Primary Health Care, Monash University, Melbourne, Australia; 9 Department of Family and Community Medicine, University of Toronto, Toronto, Ontario, Canada; 10 Centre for Research on Inner City Health and Department of Family and Community Medicine, St. Michael's Hospital, Toronto, Ontario, Canada; 11 Department of Health Policy, Management and Evaluation, University of Toronto, Toronto, Ontario, Canada

**Keywords:** Performance measurement, Primary care, Quality of care, Evaluation

## Abstract

**Purpose:**

To evaluate the appropriateness of potential data sources for the population of performance indicators for primary care (PC) practices.

**Methods:**

This project was a cross sectional study of 7 multidisciplinary primary care teams in Ontario, Canada. Practices were recruited and 5-7 physicians per practice agreed to participate in the study. Patients of participating physicians (20-30) were recruited sequentially as they presented to attend a visit. Data collection included patient, provider and practice surveys, chart abstraction and linkage to administrative data sets. Matched pairs analysis was used to examine the differences in the observed results for each indicator obtained using multiple data sources.

**Results:**

Seven teams, 41 physicians, 94 associated staff and 998 patients were recruited. The survey response rate was 81% for patients, 93% for physicians and 83% for associated staff. Chart audits were successfully completed on all but 1 patient and linkage to administrative data was successful for all subjects. There were significant differences noted between the data collection methods for many measures. No single method of data collection was best for all outcomes. For most measures of technical quality of care chart audit was the most accurate method of data collection. Patient surveys were more accurate for immunizations, chronic disease advice/information dispensed, some general health promotion items and possibly for medication use. Administrative data appears useful for indicators including chronic disease diagnosis and osteoporosis/ breast screening.

**Conclusions:**

Multiple data collection methods are required for a comprehensive assessment of performance in primary care practices. The choice of which methods are best for any one particular study or quality improvement initiative requires careful consideration of the biases that each method might introduce into the results. In this study, both patients and providers were willing to participate in and consent to, the collection and linkage of information from multiple sources that would be required for such assessments.

## Background

Primary care, the first point of contact between patients and the health care system, includes disease prevention, health promotion, and chronic disease management. For over a decade, improving primary care has been a key element of health system reform around the world to improve health and health outcomes and reduce the cost of health care [1-9] A program of research to evaluate the impacts of such reforms is essential [10,11]. The Canadian Institute for Health Information (CIHI) has proposed a comprehensive set of primary care outcome indicators [12], and also noted significant gaps in the availability and quality of the data sources to populate these indicators [13]. There is a need for a set of validated field tested measurement tools to facilitate the population of these indicators [14]. These tools could also be used for quality improvement initiatives in primary care practices and to enhance accurate reporting of performance for pay for performance incentives [15]. Administrative data, electronic health records, chart audits, patient surveys, provider surveys, population level surveys and direct observation have all been used for performance measurement in primary care, and could be used in the evaluation of primary care reform initiatives [14,16-28].

The specific aim of this study was to attempt a comprehensive measure of the quality of primary care provided by community based, multidisciplinary, primary care practices and to assess which measurement methods were best for which elements of care. This paper focuses on indicators related to the CIHI objectives on the delivery of “comprehensive” and “high quality and safe primary health care services” [12]. Five of the proposed comprehensive care indicators and fifteen proposed quality of care indicators under this objective were selected for inclusion (Table 1). Our underlying hypothesis was that the method of measurement would have a significant impact on the results obtained for many outcomes. An improved understanding of the types, magnitude, and direction of bias or error introduced by particular methods was identified as being required to aid in the appropriate selection of methods for practice level performance reporting and quality improvement activities.

**Table 1:** Indicators and potential data sources

**Indicator**	**CIHI Definition**	**Patient Survey**	**Chart Abstraction**	**Administrative Data**
Smoking rate	% of pop that are current smokers	X	X	
Smoking cessation advice	% smokers receiving advice or help on smoking cessation	X	X	
Dietary advice	% with unhealthy eating habits offered advice or help on dietary practices	X	X	
Physical Activity advice	% of inactive patients offered advice or help on regular PA	X	X	
Self Management resources	% of patients with a chronic condition provided with resources to support self management	X	X	
Influenza immunization 65+	% of patients age 65+ with influenza vaccine in the last 12 months	X	X	X
Breast cancer screening	% women age 50-69 with mammography and clinical breast exam within 2 years	X	X	X
Cervical cancer screening	% women age 18-69 with PAP smear within 3 years.	X	X	X
Colon cancer screening	% of patients age 50+ with FOBT within 24 months	X	X	X
Bone Density screening	% of women over age 65 screening with BMD at least once	X	X	X
BP testing	% of patients age 18+ who have had BP measured within 24 months	X	X	
BP control	% patients with HTN with BP <140/90	X	X	
Dyslipidemia screening	% of patients (men >40, women >55) who have had a full fasting lipid profile within 24 months	X	X	X
Dyslipidemia treatment	% patients with CAD and elevated lipids offered lifestyle advice and/or lipid lowering medication	X	X	Partial
Risk Factor screening CAD	% patients with CAD who received FBS, lipid profile, BP measurement and obesity screening within the last 12 months	X	X	Partial
Risk Factor screening HTN	% patients with HTN who received FBS, lipid profile, renal function, BP measurement and obesity screening within the last 12 months	X	X	Partial
Risk Factor Screening DM	% patients with DM who received AIC, lipid profile, nephropathy screening, BP measurement and obesity screening within 12 months	X	X	Partial
DM Glycemic control	% patients with DM with HbA1C 7.0% or less	X	X	
Vision screening in DM	% patients with DM who saw an ophthalmologist or optometrist within 24 months	X	X	X

## Methods

### Setting and participants

This cross sectional study was set in seven Family Health Teams (FHTs) in Eastern Ontario. FHTs are multidisciplinary group practices that share one of three non-fee for service funding mechanisms and receive support for information technology and integration of allied health providers into the practice^.^ A convenience sample of 7 teams was approached and all agreed to participate. Within each FHT 5-7 physicians were selected for participation as determined by local factors such as office locations and division of larger sites into functional units. In office, sequential recruitment of 20-30 patients per physician was conducted over a 9 month period in 2008. Recruitment was conducted on regular clinic days where a mix of patients were being seen. Providers and practices were not informed about which patients had agreed to participate in the study. Each participating practice, physician and associated nursing or allied health professional (AHP) was asked to complete a survey and the physicians were asked to consent to identification of their information in administrative data sets. Each patient was asked to complete a 2 part survey and consent to a chart audit and linkage of their survey and chart audit data to administrative data sets. Physician, AHP, and practice survey data was collected for measurement of other outcomes (for example team functioning) that were provided to the teams as a part of our feedback process and are reported elsewhere [29].

### Data collection

Table 1 presents a summary of the CIHI indicators selected, their definitions and the data sources that could be used to populate them. In addition, data on specific guideline related outcomes for the chronic diseases included were also collected, including detailed medication use data, neuropathy screening in DM, and control of hyperlipidemia.

#### Patient survey

This survey consisted of 2 sections. The first section was completed in the waiting room before the visit with the provider. It captured patient descriptive information and elicited patients’ experiences of the practice’s performance on measures covering a broad range of dimensions of health care service delivery. The second section was completed after the appointment with the provider and captured visit-specific information, including measures of activities related to health prevention, promotion and chronic disease management. Questions were derived from other validated survey tools, including the Primary Care Assessment Tool (PCAT-Adult), the Patient Perceptions of Patient-Centredness(PPPC), the Canadian Community Health Survey and the National Physician Survey [22-26].

#### Chart audit

The chart audit forms captured information for four thematic areas: 1) patient demographic information, 2) visit activities (including referrals, prescriptions and orders), 3) chart organization and 4) measures of performance of technical quality of care, including prevention, and chronic disease management. Chart abstractors were all provided standardized training and detailed written support material, which was based on those used in another major study of primary care models in Ontario [21]. For items such as colorectal cancer screening where multiple options for screening are available information on each individual method was collected then a calculated value for completion determined during data analysis. While all practices in this study had electronic health records (EHRs), we used a trained research associate to extract data rather than any automated data search strategy. This allowed a search of free text areas of EHRs, supplementary paper records (which were still retained or in use in many practices), scanned image files of reports, and old charts. Questions arising during a chart abstraction were emailed centrally and resolved with input from the investigators. Independent re-abstraction of a sample of 60 charts was conducted to validate the data extraction process. The discrepancies were adjudicated by a third party and all disagreements between chart abstractors were recorded and tallied. There was over 95% agreement between abstractors. The final data set was adjusted with the consensus value when discrepancies were noted.

#### Administrative billing data review

The Institute for Clinical Evaluative Sciences (ICES) is an agency supported by the government of Ontario yet operating at arms length, which is charged with analysis of health sector administrative data in Ontario. Data holdings include a number of databases with information on providers and their practices, such as physician billings, drug utilization for publicly funded prescription medications, hospital inpatient and emergency room care, and census data. Consent from participating patients and providers to access their related administrative billing data was obtained for all physicians and patients. For those measures for which administrative data was available, a performance score using this data was determined using algorithms developed for earlier studies [18]. A data set with the results for each patient was created and linked to the chart and survey data using the ICES Key Number. The ICES key number (IKN) is a unique identifier assigned based on the Ontario Health Insurance Plan(OHIP) number of the patient. Provider ID numbers at ICES are based on College of Physicians and Surgeons of Ontario registration numbers and can also be linked in similar manner. All other study data was indexed using anonymous study ID numbers. Profiles of participating physicians and practices were created to allow comparison between study patients; all patients of study physicians(to assess bias introduced by recruitment method for patients); and a larger sample of all patients of FHTs in comparable locations (All Ontario FHT patients with the exception of those in Toronto and rural areas). This final comparison was to see if our convenience sample, which included 5 academic FHTs and 2 community based FHTs from two urban areas in Ontario outside of Toronto, was similar to other FHTs in comparable locations.

## Data management and analysis

Survey and audit data were entered into SPSS version 16. Statistical analysis of comparisons between survey and chart audit data was also conducted in SPSS. Any analysis including comparisons to administrative data was conducted in SAS version 9.2. The results obtained from each data source were compared directly using both a comparison of proportion of concordant pairs and the kappa statistic. The clinical significance of the presence of discordant items, the likely reasons underlying discordant data, and the magnitude of the difference in result between methods were also considered [30,31]. To facilitate analysis we made some minor modifications to some of the definitions established by CIHI (for example looking at the individual components of composite measures, modifying time frames to match standard administrative data analysis algorithms). We did not assume that the chart was the “gold standard” for each item, using clinical experience and knowledge of the limitations of each data source to instead consider why the data sources did not always agree.

## Ethics

Ethics review and approval was obtained from the Research Ethics Boards at Queen’s University, The University of Ottawa, The Ottawa Hospital, and Sunnybrook Hospital (ICES). All study procedures underwent privacy review and approval at ICES.

## Results

Seven teams, 41 physicians, 94 associated staff and 998 patients were successfully recruited. Results from one site that kept detailed logs revealed an overall patient participation rate of 90%. Fifteen to twenty patients per day were recruited by a team of 2 research assistants. Completed surveys were returned by 813 patients (81%), 38 physicians (93%) and 77 associated staff (83%). For the items in this paper for which survey data is presented valid responses were obtained from most subjects (86%-99%). Chart audits were successfully completed on all but one patient. There was over 95% agreement between chart abstractors on the sample of charts selected for validation. Linkage to administrative data was successful for 100% of participating patients and physicians. The results of the physician, AHP, and practice surveys were not used to determine patient level outcomes and will be reported elsewhere. Table 2 outlines the socio-demographic characteristics of the study patient sample in relation to other patients from the same practices and patients from all the FHTs in Ontario. The table shows that the study participants included more female, older, sicker patients than those found in the same practices and in other Ontario FHTs.

**Table 2:** Socio-demographics of study sample

**Characteristic**	**All Study Participants**	**Patients from Participating FPs (not Study Patients)**	**All Ontario FHTs (Excluding Toronto and communities <100,000 pop)**
N = Total	998	31,223	894,230
Age	50.95 ± 19.79	42.62 ± 22.74	40.19 ± 22.74
% <16 years	5.9%	15.5%	18.0%
% > 65 years	27.2%	18.2%	15.8%
% Female	61.4%	52.3%	53.3%
Rural Location	5.8%	5.3%	6.9%
% with DM	13.8%	7.6%	7.2%
% with HTN	36.3%	22.6%	20.5%
% with prior MI	1.6%	1.2%	1.4%
% with CHF	2.5%	2.0%	2.0%
Income Quintiles	0.3%	0.2%	0.4%
Lowest 1	13.9%	15.3%	16.4%
2	19.3%	18.2%	19.1%
3	19.6%	20.7%	20.1%
4	22.6%	22.8%	22.0%
Highest 5	24.1%	22.9%	22.0%
Mean RUB*	2.7	2.1	2.1
ADG** 0-4	75.1%	86.7%	87.9%
5-9	20.8%	11.8%	10.7%
≥10	4.1%	1.5%	1.3%

*Resource Utilization Band, ** Adjusted Diagnosis Group Note: following the completion of this study a change in the methods used at ICES to identify patients allocated to physicians was introduced to correct for changes in the data received. A preliminary review of this table using the revised methods failed to demonstrate any significant differences from what is reported above. All study participants were located in urban centres other than Toronto. Rural FHTs (pop < 100,000) and FHTs from the greater Toronto area were therefore not included in this comparison.DM = Diabetes Mellitus, HTN = Hypertension MI = Myocardial Infarction CHF = Congestive Heart Failure.

TableS 3, Table 4, Table 5, Table 6 and Table 7 present the results for preventive health interventions (Table 3), health promotion (Table 4), and Chronic Disease Management (Table 6 and Table 7). For measurement of preventive health activities Table 2 shows that administrative data had both over 80% agreement with kappa statistics >.4 for mammography and BMD when compared to chart abstraction data. The kappa statistics were between .35-.45 and the levels of agreement lower (70-75%) for colorectal and cervical cancer screening, with a tendency for administrative data to underestimate rates of completion, while for immunization against influenza there was <60% agreement and a kappa of .25. The patient survey showed >75% agreement with the chart abstraction for mammography, BMD, cervical screening and clinical breast exam, but the concordance of the kappa statistic was only >.4 for BMD. There was less than 75% agreement for influenza immunization and colorectal screening with kappa values under .21 for both.

**Table 3:** Screening and preventive care (Mammography /PAP smear/ Influenza vaccination/Bone mineral density/Colorectal cancer screening) - Table of administrative data vs chart abstraction vs patient survey

**Chart Abstraction**		**Administrative Data**				**Patient Survey**			
Colorectal Screening*		Yes	No	Total	Kappa (95% CI)	Yes	No	Total	Kappa (95% CI)
	Yes	175	127	302	.442 (.372, .511)	138	105	243	.046 (-.052, .144)
50+ yrs	No	26	202	228	% agreement	87	80	167	% agreement
	Total	201	329	530	=71%	225	185	410	=53%
Bone Mineral		Yes	No	Total	Kappa (95% CI)	Yes	No	Total	Kappa (95% CI)
Density ≤5 yrs	Yes	123	6	129	.852 (.789, .916)	86	15	101	.667 (.549, .785)
65+ yrs**	No	13	115	128	% agreement	18	65	83	% agreement
	Total	136	121	257	=93%	104	80	184	=82%
Flu Shot ≤ 2 yrs*		Yes	No	Total	Kappa (95% CI)	Yes	No	Total	Kappa (95% CI)
65+ yrs	Yes	83	102	185	.253 (.168, .338)	133	15	148	.209 (.056, .353)
	No	9	71	80	% agreement	40	16	56	% agreement
	Total	92	173	265	=58%	173	31	204	=73%
PAP ≤ 2 yrs*		Yes	No	Total	Kappa (95% CI)	Yes	No	Total	Kappa (95% CI)
Females	Yes	255	100	355	.351 (.265, .437)	270	12	282	.232 (.092, .372)
18-69 yrs	No	14	57	71	% agreement	42	12	54	% agreement
	Total	269	157	426	=73%	312	24	336	=84%
Clinical Breast Exam ≤ 2 yrs*		Yes	No	Total	Kappa (95% CI)	Yes	No	Total	Kappa (95% CI)
						118	16	134	.094 (-.09, .278)
Females			*Not	in	Billing*	19	5	24	% agreement
50-69 yrs						137	21	158	=78%
Mammogram* ≤ 2 yrs		Yes	No	Total	Kappa (95% CI)	Yes	No	Total	Kappa (95% CI)
	Yes	146	20	166	.407 (.241, .573)	135	4	139	.190 (-.012, .392)
Females	No	13	17	30	% agreement	20	4	24	% agreement
50-69 yrs	Total	159	37	196	=83%	155	8	163	=85%

*Comparing survey “ever” done to CA search back in chart for 2 yrs.

**Comparing survey “ever “done to CA search back in chart for 5 yrs.

Note that the total number of subjects for each item varies based on eligibility for the manoeuvre as well as completeness of the data set. Chart abstraction data had very few missing values as for most items missing was coded as “No”. However, in the patient survey some individual questions or sections of the survey were skipped resulting in a reduced subsample for the comparison in a given question.

**Table 4:** Health promotion (Diet/exercise/smoking status) - Table of chart abstraction vs patient survey

**Chart Abstraction**		**Patient Survey**			
Diet		Yes	No	Total	Kappa (95% CI)
	Yes	126	46	172	.178 (.046, .310)
	No	5	10	15	% agreement
	Total	131	56	187	=73%
Exercise		Yes	No	Total	Kappa (95% CI)
	Yes	90	33	123	.194 (.030, .358)
	No	10	12	22	% agreement
	Total	100	45	145	=70%
Ever Smoked		Yes	No	Total	Kappa (95% CI)
	Yes	101	177	278	-.468 (-.544, -.404)
	No	181	37	218	% agreement
	Total	282	214	496	=28%
Smokers Still		Yes	No	Total	Kappa (95% CI)
Smoking	Yes	73	23	96	.722 (.64, .804)
	No	17	297	314	% agreement
	Total	90	320	410	=90%

**Table 5:** Chronic disease status/Management between the chart abstraction and both administrative data and the patient survey

**Chart Abstraction**		**Administrative Data**				**Patient Survey**			
Has Diabetes		Yes	No	Total	Kappa (95% CI)	Yes	No	Total	Kappa (95% CI)
	Yes	125	6	131	.863 (.817, 908)	84	14	98	.860 (.800, .920)
	No	27	777	804	% agreement	9	573	582	% agreement
	Total	152	783	935	=96%	93	587	680	=97%
Has Hypertension		Yes	No	Total	Kappa (95% CI)	Yes	No	Total	Kappa (95% CI)
	Yes	298	40	338	.752 (.709, .796)	188	71	259	.689 (.631, .747)
	No	69	528	597	% agreement	26	404	430	% agreement
	Total	367	568	935	=88%	214	475	689	=86%
Has CAD						Yes	No	Total	Kappa (95% CI)
	Yes	Not	in	ICES	Data	36	25	61	.653 (.543, .763)
	No					9	610	619	% agreement
	Total					45	635	680	=95%
Anti-hypertensive		Yes	No	Total	Kappa (95% CI)	Yes	No	Total	Kappa (95% CI)
Medications	Yes	146	47	193	.557 (.459, . 655)	115	48	163	.551 (.449, .653)
65+ yrs	No	9	68	77	% agreement	5	69	74	% agreement
	Total	155	115	270	=79%	53	184	237	=78%
Anti-lipidemic		Yes	No	Total	Kappa (95% CI)	Yes	No	Total	Kappa (95% CI)
Medications	Yes	81	104	185	.230 (.143, . 316)	59	21	80	.611 (.483, .739)
65 + yrs	No	12	73	85	% agreement	8	60	68	% agreement
	Total	93	177	270	=57%	67	81	148	=80%

**Table 6:** Comparison of chronic disease management (CDM) between administrative data and patient survey

**Administrative Data**		**Patient Survey**				
Anti-hypertensive		Yes	No	Total	Kappa	95% CI
Medications	Yes	83	49	132	.426	(0.315, .537)
65+ yrs	No	20	85	105		% agreement
	Total	103	134	237		=71%
Anti-lipidemic		Yes	No	Total	Kappa	95% CI
Medications	Yes	40	39	79	.349	(0.222, .476)
65 + yrs	No	27	131	158		% agreement
	Total	67	170	237		=72%

**Table 7:** Comparison of chronic disease management (CDM) between chart abstraction and patient survey

**Chart Abstraction**		**Patient Survey**				
Was any advice/info		Yes	No	Total	Kappa	95% CI
/resources/websites	Yes	280	26	306	.39	(0.346, .434)
/pamphlets given	No	359	580	939		% agreement
regarding disease?	Total	639	606	1245		=69%
Patient ever had a heart		Yes	No	Total	Kappa	95% CI
attack?	Yes	14	3	17	0.531	(.355, .708)
	No	18	221	239		% agreement
	Total	32	224	256		=92%
Hospitalized for		Yes	No	Total	Kappa	95% CI
Chronic disease	Yes	6	6	12	0.385	(.149, .621)
	No	11	275	286		% agreement
	Total	17	281	298		=94%
Wt/Ht/Wc ≤12mo?		Yes	No	Total	Yes	No
	Yes	188	33	221	0.166	(.500, .272)
	No	82	35	117		% agreement
	Total	270	68	338		=66%
Fasting Blood		Yes	No	Total	Kappa	95% CI
Sugar^a^	Yes	223	20	243	0.122	(0, .244)
	No	54	12	66		% agreement
	Total	277	32	309		=76%
Lipid profile done^b^		Yes	No	Total	Kappa	95% CI
	Yes	220	6	226	0.312	(.122, .511)
	No	22	8	30		% agreement
	Total	242	14	256		=89%
BP controlled^c^ (<140/90) ^d^		Yes	No	Total	Kappa	95% CI
	Yes	109	10	119	0.218	(.086, .35)
	No	50	19	69		% agreement
	Total	159	29	188		=68%
EKG^c^		Yes	No	Total	Kappa	95% CI
	Yes	31	11	42	0.298	(.168, .428)
	No	49	93	142		% agreement
	Total	80	104	184		=67%
Renal function ^e^		Yes	No	Total	Kappa	95% CI
(microalbumin)	Yes	54	4	58	0.023	(-.151, .204)
	No	21	2	23		% agreement
	Total	75	6	81		=69%
Lipids controlled		Yes	No	Total	Kappa	95% CI
(LDL < 4.5 &	Yes	101	7	108	0.337	(.087, .587)
total chol :HDL < 6.0)^f^	No	10	6	16		% agreement
	Total	111	13	124		=86%
AIC ≤2 yrs ^e^		Yes	No	Total	Kappa	95% CI
	Yes	41	5	46	-.117	(-.189,.045)
	No	6	0	6		% agreement
	Total	47	5	52		=79%
Foot exam ^e^		Yes	No	Total	Kappa	95% CI
	Yes	51	3	54	0.327	(.103, .551)
	No	16	8	24		% agreement
	Total	66	11	78		=76%
Eye exam ^e^		Yes	No	Total	Kappa	95% CI
	Yes	53	4	57	0.15	(-.074, .374)
	No	17	4	21		% agreement
	Total	70	8	78		=73%
ASA		Yes	No	Total	Kappa	95% CI
	Yes	92	5	97	0.572	(.472, .672)
	No	45	85	130		% agreement
	Total	137	90	227		=78%
ACE		Yes	No	Total	Kappa	95% CI
	Yes	93	14	107	0.722	(.622, .822)
	No	13	78	91		% agreement
	Total	106	92	198		=86%
ARB		Yes	No	Total	Kappa	95% CI
	Yes	37	13	50	0.703	(.583, .823)
	No	8	128	136		% agreement
	Total	45	141	186		=89%
Beta blocker		Yes	No	Total	Kappa	95% CI
	Yes	58	10	68	0.675	(.568, .783)
	No	20	110	130		% agreement
	Total	78	120	198		=85%
Calcium channel		Yes	No	Total	Kappa	95% CI
blocker^g^	Yes	36	11	47	0.68	(.552, .808)
	No	10	101	111		% agreement
	Total	46	112	158		=87%
Diuretic^g^		Yes	No	Total	Kappa	95% CI
	Yes	86	11	97	0.701	(.689, .813)
	No	13	56	69		% agreement
	Total	99	67	166		=86%
Statin		Yes	No	Total	Kappa	95% CI
	Yes	113	95	208	0.005	(-.045, .055)
	No	3	3	6		% agreement
	Total	116	98	214		=54%

Notes: ^a^ Survey asked if ever; CA reported < = 2 y. ^b^Survey < 12 mo; CA < = 2 y. ^c^Specific to HTN only. ^d^ 2 high risk patients with BP over low risk cut offs but under high risk cut offs, correctly identified themselves as not under control. ^e^ Specific to DM only. ^f^2 high risk patients with lipids over low risk cut offs but under high risk cut offs, correctly identified themselves as not under control. ^g^Asked under HTN section in survey only.

Table 4 outlines the agreement levels observed in health promotion activities between the patient survey replies and the information found in their chart only as this information is not available through billing records. The only one that had concordance over 75% was current smoking status. Past smoking status showed less than 50% agreement and provision of advice on diet and exercise showed 70-75% concordance with kappa levels all <.2. The levels of agreement between the administrative and chart data as well as between the patient survey and the chart are shown for the presence of the index conditions of interest and use of two broad categories of medications are presented in Table 5. Table 6 supplements this with a comparison between the survey data and administrative data for medication use on patients over 65. There was >85% agreement on the presence of the diagnosis. For medication use, concordance between the chart data and patient surveys was >75%, while the concordance between administrative and chart data was >75% for antihypertensives and only 57% for anti-lipidemics, with much higher rates of lipid lowering agent use noted in the charts. In Table 6 the level of agreement between administrative data and the patient between 70-75%, which is slightly lower than level of agreement between the chart and the patient. The kappa statistic was higher for antihypertensives, than for anti-lipidemic drugs.

Table 7 presents the comparison for a number of disease specific recommendations for the chronic diseases we examined, including a more detailed review of medication usage. Documentation of advice or resources provided has a level of agreement of <70%, with fewer than half of the events reported by patients being noted in the chart and a kappa <.4. For relatively less common but important key events such as MI and hospitalization, while agreement was >90% overall, there was poor agreement between the chart and patients who report having had these events, with less than half being identified in the charts. For the remaining process of care measures there was a mixed result, with levels of agreement being >75% for FBS, Lipid profiles, control of hyperlipidemia, AIC and foot exams and <75% for the remainder. Kappa values were <.4 for all process of care outcomes other than medications. The more detailed medication profiles showed levels of agreement >85% with kappas >.6 with the exception of ASA and Statins. ASA is an over the counter drug and despite a 78% agreement rate and kappa >.4, was only recorded in the chart in about 2/3 of patients who reported using it. In contrast, statins had only 54% agreement and a kappa <.2 due to patients reporting not taking them despite the drug being noted as active in their chart.

## Discussion

There are relatively few studies in primary care performance measurement which have examined the differences in results obtained through different methods of measurement, especially involving administrative data [17,20,32,33]. This study examined the validity of data on primary care performance indicators obtained by various methods, as well as the acceptability, feasibility and potential biases of using a practice-based recruitment approach for the collection of linked data from a range of different methods. Our ability to collect data on multiple measures by audit, survey, and use of administrative data with good participation rates, high agreement rates between chart assessors, high rates of completion for surveys, and little objection to data linkage, shows that the collection of linked data from multiple sources is both acceptable and feasible. This study used sequential recruitment of patients presenting for care in participating practices [22]. Participants had 50-100% higher rates of chronic disease and multi-morbidity than the practice population, children were under-represented, and women and the elderly were overrepresented. Studies seeking a representative sample of all practice patients may wish to use other methods of recruitment,.Studies on the care experiences of chronic disease patients, workload or work process issues or the daily experiences of practitioners may find this to be both appropriate and efficient. As our study was focused on the concordance between data collection methods this design was unlikely to impact our results.

Significant differences in performance were found using the different data collection methods for many indicators. No single data collection method emerged as consistently the most valid across all performance indicators. A only a limited number of indicators had kappa statistics >.4 (moderate or better agreement) [30] however in come cases these were much worse than the degree of concordance estimated by proportion of concordant pairs. When interpreting our data both kappa and the degree of concordance should be considered [31]. With the increasing use of administrative data for primary care performance reporting [19,34,35], remuneration and funding decisions [28], disease registries [27] and public health reporting [36] the comparison of administrative data results to multiple different data sources is especially important for guiding the use and interpretation of administrative data results in future research, policy, and planning [27,37,38].

Good agreement across measurement methods was seen for preventive health imaging such as mammograms and bone mineral density scans. Previous research has found similar mammogram screening rates using patient surveys and chart audits [17,32]. This study adds the use of administrative data to this comparison and finds it to be a reasonable alternative. Notable areas of discordance between administrative data and other methods of data collection included Pap smears, colon cancer screening and influenza vaccination, all of which had much lower rates noted in the administrative data. Manoeuvres like Pap tests and colon cancer screening may be completed in contexts other than the delivery of primary care. Where services that are not included in routine administrative data sources, and recourse to them is more widespread administrative data may be less accurate in capturing care received by the patient than other methods. Thus, the local context of care may significantly alter the validity of administrative data results for primary care performance.

Important differences between patient survey reports and chart audits were also found. Pap smears and influenza vaccination were reported at higher rates in the surveys. Patient over-reporting of Pap test rates has been previously reported [32]. Influenza vaccination in Ontario often occurs in public flu clinics and is therefore not always noted in the chart, so higher rates would be expected for this outcome. Patients who report having received diet and exercise advice had a notation to this effect in their chart over 90% of the time. The more significant difference was in the opposite direction, with 25-30% of patients whose charts indicated this was discussed failing to report this in the survey. This finding contradicts previous research comparing chart audit and patient survey to actual observation of patient visits, which found patients reported receiving advice more frequently than was noted in the chart [17]. This could be due in part to the multidisciplinary nature of these family health teams, where allied health providers would be contributing to the delivery of these services and documentation of this in the patient record. However, this finding may also reflect the difficulties in communicating messages on health promotion in ways that are memorable or retained by patients.

For most medications there was good agreement between chart and survey responses, and moderate agreement between survey and administrative data. There were two exceptions. For statins agreement between the chart and survey was poor, while there was much better agreement with administrative data. These discrepancies may represent issues of medication adherence, or poor understanding or recognition of a medication. Aspirin, which is available over the counter (OTC), was reported more frequently by patients than noted in the charts, likely representing lack of documentation in the chart. These findings suggest that patient survey may be the more accurate source for medications used, however this source is not without its potential biases. In terms of clinical outcomes, patients were more likely to report that their blood pressure and lipids were at target than was reported in the chart. These findings highlight the importance of importance of clear communication between patients, their physicians and other providers on issues such as medication use and management targets.

Recent efforts to assess the quality of chronic disease management (CDM) has relied on administrative data, EHR audits and population based surveys to identify the patient population with the condition of interest [36,38]. For identifying the population with diabetes, there was strong agreement across measurement methods. For hypertension, the level of agreement was still good, but not as much as for diabetes. For estimation of prevalence in the sample the margin of difference is fairly small (39% vs 36%), but there are large numbers of discordant pairs. In these situations, administrative data identify more cases than in the charts, perhaps a reflection of care that occurs in specialist or hospital settings and not captured in the primary care chart. For the purposes of registry generation, either method would be appropriate, with the cost of administrative data being much lower. Performance measurement in primary care increasingly forms the basis of quality improvement investments, performance bonuses, population health planning and reporting [15]. Others have expressed concerns about the unintended consequences of pay for performance systems and on the impact of performance measurement more generally on good clinical practice. [38,39] Our results indicate that careful consideration needs to be given to the methods used for assessing performance if these concerns are to be minimized. Future performance reporting should account for potential bias in results based on the data collection method and indicators measured.

### Limitations

The study was carried out in only one region of Ontario, Canada, using a convenience sample of practices that included a large number of academic teaching practices. Many of these practices used hospital labs, reducing the ability of administrative data to capture those tests. The structure and quality of records may impact the results of the chart audit and may not be reflective of chart content in other locations. Ontario has extensive administrative data sets that have been cleaned for use in health services research studies and an extensive program of development of programming expertise and algorithm development that may not be available in all jurisdictions. Patients in Canada may also have different attitudes towards data privacy and linkage than others than those in other countries. We applied rules for eligibility for manouvers based on age, sex, presence of index conditions and also assessed for any exclusion criteria within existing guidelines, but did not attempt to determine reasons for non-completion (ie. Patient refused, deliberate deviation from guidelines due to co-morbidities etc…). In addition, due to concerns with patient recall about timing of specific manouvers we elected to use an “ever received” format for our survey questions. The potential bias introduced would increase the degree of discrepancy observed and may partially explain the lower degree of agreement seen between the patient survey and chart abstraction for colorectal screening, which for most patients in Ontario is conducted with an annual FOBT. In any study there is the possibility of a Hawthorne effect, however in this study it is not likely as providers were not aware of which patients were participating and data collection was retrospective.

## Conclusions

For many measures of technical quality of care, chart audit remains the most accurate method of data collection. Patient surveys are required for more accurate assessment of indicators such as immunizations, chronic disease advice/information dispensed, medication use, and some general health promotion items. Consecutive sampling of patients in the waiting room samples a population that is sicker, older and more likely to be female compared to the practice population. Administrative data appears useful for a number of indicators including several aspects of screening and chronic disease diagnosis. Administrative data are much less costly than other methods of data collection and can cover entire populations. Recruitment rates of physicians and patients remained high while requesting permission to link the data collected at the practice to the provincial health administrative databases. A comprehensive understanding of primary care performance will require the use of multiple data collection methods for the foreseeable future. The choice of which methods are best for any one particular study or quality improvement initiative requires careful consideration of the biases that each method might introduce into the results. Future studies should also consider assessing the reasons underlying divergence between decisions made at the individual patient level and recommended guidelines.

## Abbreviations

FHT, Family Health Team; FFS, Fee for Service; CIHI, Canadian Institute for Health Information; ICES, Institute for Clinical Evaluative Sciences; BMD, Bone Mineral Density; FOBT, Fecal Occult Blood Test; FOBT, Fecal Occult Blood Test; PAP, Papanicolaou Smear.

## Competing interest

None of the authors have any conflicts of interest to report.

## **Authors’ contributions**

MEG and WH conceived the study. All authors contributed to study design. MEG, WH, SJ and CS were responsible for primary data collection. RJL, RHG and MEG were responsible and CS for the administrative data analysis. MEG and CS directed the initial data analysis. All authors contributed to decisions on the interpretation of results and contributed to the drafting and of the manuscript. All authors approved the version of the manuscript prior to submission.

## Prior Presentations

Portions of this manuscript content have been presented at the Canadian Institutes for Health Research Primary Care Summit, Toronto, Jan 18, 2010, the Canadian Association for Health Services and Policy Research Annual Meeting, Calgary May 12, 2009 and the North American Primary Care Research Group, San Juan Puerto Rico, Nov 16, 2008.

## Support

This project was funded by a grant from the Ontario Ministry of Health and Long Term Care. ICES and the Centre for Health Services and Policy Research are also supported in part by the Ontario Ministry of Health and Long Term Care. The opinions, results and conclusions reported in this paper are those of the authors and are independent from the funding sources. No endorsement by ICES or the Ontario MOHLTC is intended or should be inferred.

## Pre-publication history

The pre-publication history for this paper can be accessed here:

http://www.biomedcentral.com/1472-6963/12/214/prepub
